# Effect of Sasobit/Waste Cooking Oil Composite on the Physical, Rheological, and Aging Properties of Styrene–Butadiene Rubber (SBR)-Modified Bitumen Binders

**DOI:** 10.3390/ma16237368

**Published:** 2023-11-27

**Authors:** Xiongfei Zhao, Zhen Lu, Hengyu Su, Qiaoli Le, Bo Zhang, Wentong Wang

**Affiliations:** 1 Infrastructure Department, Guizhou Minzu University, Guiyang 550025, China; zhaoxiongfei199@163.com; 2 School of Civil Engineering and Architecture, Guizhou Minzu University, Guiyang 550025, China; suhygzmd@163.com (H.S.); leqiaoli3@163.com (Q.L.); zhangbo_dzs@126.com (B.Z.); 3 Key Laboratory of Karst Environmental Geological Hazard Prevention, State Ethnic Affairs Commission, Guiyang 550025, China; 4 Key Laboratory of Urban Underground Space Development and Safety in Karst Areas, Guizhou Minzu University, Guiyang 550025, China; 5 College of Transportation, Shandong University of Science and Technology, Qianwan Harbor Road, Qingdao 266590, China

**Keywords:** Sasobit/WCO composite, rheological property, SBR-modified bitumen, aging property

## Abstract

The modifying effects of polymer on bitumen low-temperature performance are substantially compromised by the thermal breakdown of styrene–butadiene rubber (SBR) polymer during bitumen mixture production operations. The efficacy of the utilization of Sasobit/waste cooking oil (Sasobit/WCO) as a warm-mix additive has been demonstrated in mitigating the adverse consequences of thermal aging on SBR-modified bitumen binder (SB) while preserving the binder’s original performance characteristics. However, few studies have been conducted to further investigate the rheological properties and aging resistance of SB modified with Sasobit/WCO compounds. In this work, three additives—Sasobit, WCO, and Sasobit/WCO composite—were selected, and their effects on the physical and rheological characteristics of SB as well as the temperatures at which the mixtures were prepared were assessed. In addition, by using dynamic shear rheometers (DSR) and bending beam rheometers (BBR), the effects of this innovative warm-mix addition on the performance grade (PG) and aging resistances of SB were evaluated. According to the results, Sasobit/WCO composites outperform Sasobit and WCO in lowering the mixture preparation temperature. Sasobit/WCO also improves both the high- and low-temperature performance of SB simultaneously. Compared to hot-mix asphalt mixtures, the addition of Sasobit/WCO reduces the preparation temperature of the bitumen mixtures by 19 °C, which in turn helps to minimize the negative effects of temperature aging on the functioning of the SB. Additionally, the Sasobit/WCO composite addition can improve the SB mixture’s resistance to thermal cracking. After the introduction of Sasobit/WCO, the high-temperature PG of SB was raised by two levels, regardless of whether the warm-mix impact was taken into account. With the addition of Sasobit/WCO, SB’s resilience to short-term aging was enhanced.

## 1. Introduction

Bitumen binder (BB), which has a high adhesive capacity, is presently a widely used binding substance in pavement construction. However, the increased brittleness and decreased flexibility of BB within a climate with cold temperatures causes bitumen pavement to crack [1,2]. Due to the volatilization of light components and the formation of asphaltenes, cracking damage becomes increasingly obvious with age [3,4]. Therefore, bitumen modification is essential to enhancing bitumen pavement’s low-temperature performance, which is key to promoting sustainable pavement construction. In comparison to other polymer modifiers, styrene–butadiene rubber (SBR) is more effective at enhancing BB’s low-temperature performance [5,6]. Due to entanglements between the polymer chains of SBR, modified BB can develop a robust cross-linking network with the appropriate dosage of SBR, which can considerably increase the binder’s toughness and tensile performance at low temperatures [7,8]. However, when the SBR-modified bitumen binder (SB) combination is produced in a high-temperature environment, significant oxidation and degrading reactions of SBR are unavoidable, which can obviously weaken the promotion of SBR for thermal cracking resistance of the modified BB [9,10]. For instance, the low-temperature ductility of SB decreased by 130 cm after short-term aging [11]. In the meantime, SBR modifier’s ability to disintegrate in an oxygen-rich, high-temperature environment greatly jeopardizes the benefits of SBR on the low-temperature performance of bitumen binder due to SBR [6,12]. To solve these issues, SB’s aging and rutting resistances need to be improved. This can be achieved by adding additives that work either chemically or physically to optimize SB properties.

Warm-mix asphalt mixtures (WMA), which contain a variety of warm-mix additives, are effective in reducing the degree of BB’s short-term aging since their manufacturing temperature is 10 °C to 30 °C lower than conventional hot-mix asphalt mixtures [13,14]. The WMA technique is thus preferred to reduce SBR decomposition due to high-temperature mixing during the creation of SB mixtures. Warm-mix technologies nowadays can be roughly categorized into three groups: those that use organic additives with a viscosity-reducing effect, like Sasobit; those that change surfactants, like Evotherm; and those that use foaming technology [15,16,17,18]. Sasobit, one of the most widely used organic additives, lowers the mixing temperature of bitumen mixtures, enhancing the thermal resilience of BBs and, most importantly, lowering the risk of thermal aging [8,19,20]. For example, incorporating 4% Sasobit into the SB mixture can lower the blending temperature from 170 °C to 156 °C while elevating the SB softening point by 34.8 °C [21]. However, at 5 °C, SB’s ductility dropped precipitously from 63.5 cm to 2.5 cm. This is because the synthetic hard wax that makes up the majority of Sasobit has poor deformation properties at room temperature, substantially reducing the ductility of BB [13,20,22]. Therefore, adding Sasobit separately can simultaneously increase SB’s thermal stability and reduce the risk of aging at the expense of significantly degrading its low-temperature performance.

Due to its outstanding fluidity and thermal stability, waste cooking oil (WCO) can react with polymers to increase the flexibility of organic materials [23,24,25]. Additionally, several research studies examined how WCO affects recycled bitumen; the results indicate that WCO can increase the uniform dispersion of bitumen and the diffusibility of the rejuvenator [26,27,28,29]. The reason for this phenomenon is that by supplementing the lightweight components, the colloidal structure within the bitumen can be balanced, which improves the low-temperature ductility of BBs [30,31], Whereas the inclusion of WCO would compromise the thermal stability of BBs, and this unfavorable effect grows as WCO content is increased [32,33].

Based on the aforementioned research, the researchers found that Sasobit helped lower the mixing temperature of SB mixtures; in addition, it can be beneficial to improve the high-temperature stability and aging resistance of mixtures while significantly degrading their low-temperature performance. Adding modifiers to make up for the shortcomings of Sasobit-modified bitumen is a good option. However, WCO has the advantage of increasing the ductility of bitumen while reducing its high-temperature performance. Therefore, given the respective characteristics of Sasobit and WCO, the composite modifier is prepared by complementing the properties of both so that it can maintain the low-temperature performance of SB and at the same time improve the aging resistance and thermal stability of SB. In order to reduce the preparation temperature of SB composites, Sasobit was chosen as a typical warm-mixing component, and WCO was incorporated to compensate for the damage caused by the low-temperature performance of Sasobit.

This study’s primary goal is to reduce the mixing temperature of bitumen mixture using warm-mixing procedures to lessen the negative impacts on the properties of SB. The second is to look at how Sasobit/WCO affects SB’s rheological characteristics and aging resistance during short- and long-term aging. Based on this objective, this study firstly analyzes and compares the physical and rheological properties of SB after Sasobit, WCO, and composite modification. Secondly, the creep stiffness and viscoelasticity characteristics of SB at different aging temperatures are tested using BBR and DSR tests to evaluate its aging resistance.

## 2. Materials and Methods

### 2.1. Raw Materials

#### 2.1.1. Bitumen

SK-70# produced by the Korea Bitumen Factory (Incheon, Republic of Korea) was used to prepare the modified bitumen, and Table 1 lists its physical characteristics.

#### 2.1.2. SBR

An anionic polymer modifier called liquid latex SBR was purchased from a local factory for use in this study. The basic SBR indicators are presented in Table 2.

#### 2.1.3. Sasobit

Sasobit, a warm-mixing agent purchased from the Dalian Hongze Petrochemical Co. in Dalian, China, was used to prepare the modified bitumen samples. Since Sasobit has a melting point of roughly 100 °C, adding it to bitumen at a high temperature will cause it to evenly spread, lowering the bitumen’s viscosity. Table 3 lists the physical characteristics of the Sasobit.

#### 2.1.4. WCO

The WCO was came from local fried barbecue shops in Guiyang. Before the test, the WCO was first placed in an oven for 1.5 h to remove excess water. Then, a funnel and filter paper were used to filter out impurities and metal particles from the used oil. The physical properties of the WCO are shown in Table 4.

### 2.2. Preparation Processes of Modified BBs

**Figure 1:**
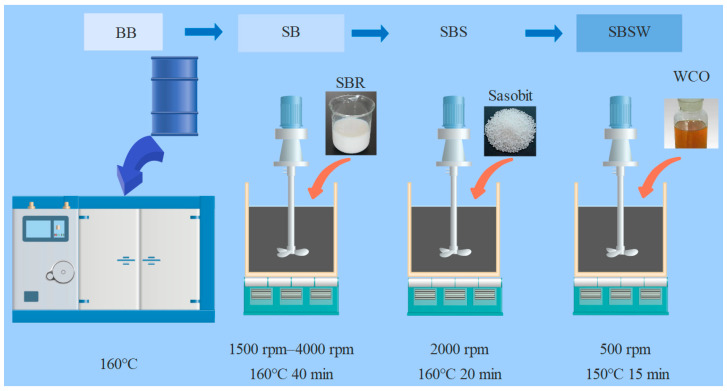
The preparation procedures of different modified bitumen samples.

The most effective ways to prepare SB and SBSW are identified in accordance with earlier research as follows [8,20,21]. The base BB was first heated at 160 °C. Then the heated base BB was slowly mixed with 4 wt% of SBR at a low speed of around 1500 rpm. SB was produced by adding all the SBR liquid to the BB and shearing at 160 °C for 40 min at a controlled speed of 4000 rpm. Following that, the mixture of Sasobit with a 4% dosage and SB was sheared at 2000 rpm for 20 min while the temperature was maintained at 160 °C. The stirring temperature was then reduced to 150 °C, and 6 wt% WCO was added. The mixture was stirred mechanically at 500 rpm/min for 15 min to produce the SBSW specimen. Figure 1 depicts the method used to prepare several modified bitumen samples. Other bitumen samples went through the identical preparation processes as SB modified by Sasobit/WCO aside from the change in the type of additives. Finally, four different types of bitumen samples—SB, SB modified only by Sasobit, SB modified only by WCO, and SB modified by a composite of Sasobit/WCO—were created. They are referred to as SB, SBS, SBW, and SBSW, respectively, for ease of expression.

### 2.3. Physical Property Tests

To evaluate the physical properties of modified BB, ductility, softening point, and penetration tests were carried out in accordance with the specifications of ASTM D36 [34], D5 [35], and D113 [36]. In addition, the rotational viscosity at 120, 135, 150, 165, and 180 °C were tested in order to construct viscosity–temperature curves and determine the mixing and compaction temperatures. In this study, three sets of samples were used for the replication of the experiment.

### 2.4. Aging Tests

When modified BB is combined with aggregates, the short-term aging temperature for BB is currently set at 163 °C, regardless of whether the test is TFOT or RTFOT. Due to the variability of materials, this temperature does not truly reflect the degree of aging of bitumen in service [37,38]. In contrast, it is more reasonable to choose the mixture’s mixing temperature as the BB’s short-term aging temperature. As stated by the equiviscosity principle, the temperature at which the bitumen has a viscosity value of 0.17 Pas is chosen as the reference temperature for the short-term aging (RTFOT, 85 min) of bitumen (based on workability).

In accordance with ASTM D6521 [39], a pressure aging vessel (PAV) test was used to simulate long-term aging for all modified BB under testing conditions of 100 °C and 2.1 MPa for 20 h. It is important to note that tests of both standard and equiviscous short-term aging temperatures were conducted to assess the PG and aging resistance of modified BB.

### 2.5. Low-Temperature Thermal Cracking

The bending beam rheometer (BBR) test was used to assess the low-temperature anticracking capability of SB, SBS, SBW, and SBSW in different aging states by ASTM D6648 [40]. Specifically, when the loading period was equivalent to 60 s, the creep stiffness (S) and relaxation property (m) of the bitumen were established. Additionally, this test also used the S/m-value ratio as a critical assessment factor. Better thermal cracking resistance is indicated by smaller S/m-values and larger m-values.

### 2.6. Temperature Sweep and Frequency Sweep Tests

All bitumen samples’ viscoelastic characteristics were determined using DSR following ASTM D7175 [41]. The temperature sweep test was conducted to assess the rheological properties. The rutting factor (G*/sinδ) was measured at 40 °C, 45 °C, 50 °C, 55 °C, 60 °C, 65 °C, 70 °C, 75 °C, 80 °C, and 85 °C. The frequency sweep tests were performed in a 0.1∼10 Hz frequency range. The test was conducted using a 25 mm plate with a control gap of 1 mm. In order to guarantee that the modified BB can always maintain a linear viscoelastic condition, the 10% strain was controlled during this test.

## 3. Results and Discussion

### 3.1. Physical Properties Test

**Figure 2:**
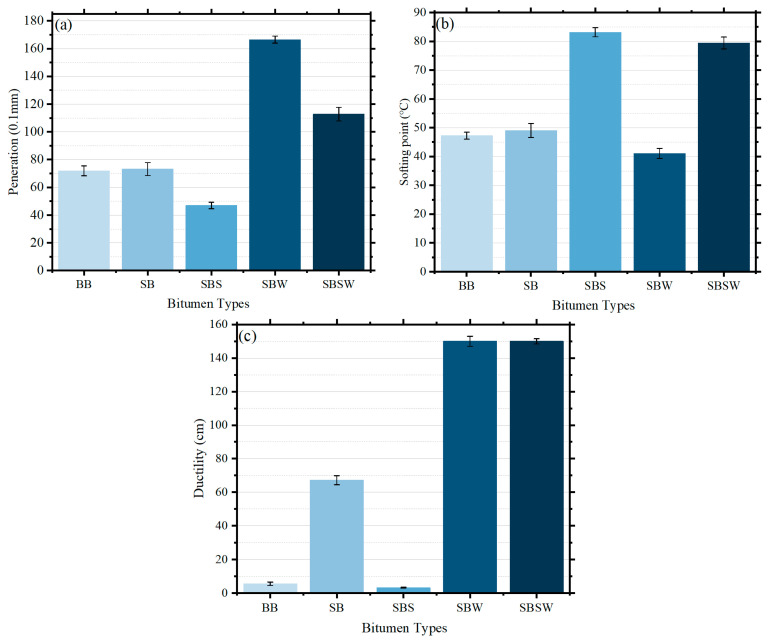
Impact of different modifiers on physical properties of SB: (**a**) penetration at 25 °C; (**b**) softening point; and (**c**) ductility at 5 °C.

All modified bitumen’s ductility, penetration, and softening point are displayed in Figure 2. The error bars in the figure represent the degree of dispersion of the needle penetration, softening point, and ductility, respectively. It is evident that with the exception of ductility, there is virtually little difference between BB and SB in terms of penetration and softening point. After the addition of Sasobit, the penetration and ductility of the modified bitumen significantly decreased, while the softening point was enhanced. Specifically, the addition of Sasobit reduces the ductility of SB at 5 °C by 63.9 cm. Therefore, adding Sasobit to SB alone would not be appropriate. The results are also similar to previous studies [20]. Nonetheless, WCO introduction leads to an 8 °C reduction in the softening point of SB, significantly compromising its high-temperature stability. In contrast, the combination of Sasobit and WCO not only elevates SB’s softening point by 30.4 °C but also enhances its ductility at 5 °C, increasing it from 67.2 cm to over 150 cm. This underscores the ability of Sasobit/WCO to enhance SB performance across a broad temperature range, spanning both high and low extremes. One may conclude that the composite of Sasobit/WCO is significantly superior to the other two additions because it can simultaneously improve SB’s rutting and cracking resistance while lowering the preparation temperatures of the SB mixture [42,43].

### 3.2. Measurement of Short-Term Aging

**Figure 3:**
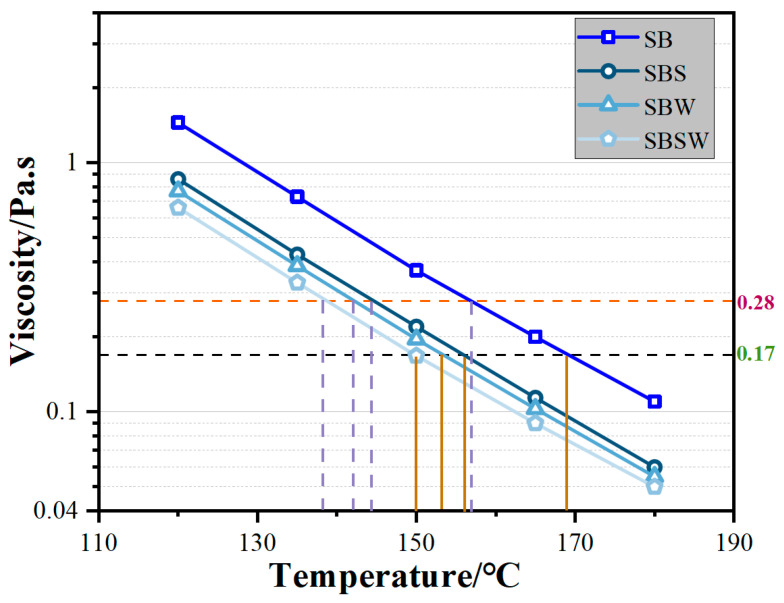
The viscosity–temperature curves for SB, SBS, SBW and SBSW.

The viscosity–temperature curves for SB, SBS, SBW, and SBSW are shown in Figure 3. The phenomenon that the other three modified SBs all exhibit viscosity values that are significantly lower than SB at 120, 135, 150, 165, and 180 °C indicates that additives may all effectively reduce the viscosity of SB. Two theories could explain this trend. First, Sasobit is recognized as a conventional warm-mix additive, attaining warm-mixing effects through viscosity reduction in the binder [44]. Second, the primary constituent of WCO consists of light oil, which can be blended with bitumen to enhance its softening by augmenting the proportion of lighter components within the bitumen. Additionally, it also serves to reduce the bitumen’s viscosity [45]. Various types of additives contribute differently to the viscosity-reducing effect of bitumen, leading to the following ranking of modified bitumen: SBS > SBW > SBSW.

The optimum mixing and compaction temperatures for bitumen correspond to temperatures at viscosity values of 0.17 Pa s and 0.28 Pa s, respectively. Both of these temperatures offer a more accurate description of the bitumen’s viscosity characteristics as it is being manufactured [46]. Table 5 displays the preparation temperatures for the various modified BBs. It is clear that SB has the greatest mixing and compaction temperatures, indicating that additives can significantly lower SB’s preparation temperatures. The Sasobit/WCO composite modifier reduced the bitumen preparation temperature by 19 °C, followed by WCO and Sasobit by 15 °C and 13 °C, respectively. It is important to note that this study conducted short-term aging tests on various modified bitumen samples at the recommended preparation temperatures in addition to the conventional short-term aging temperatures. This comprehensive approach aimed to thoroughly evaluate the aging resistance of the modified bitumen.

### 3.3. Evaluation of Low-Temperature Performance

**Figure 4:**
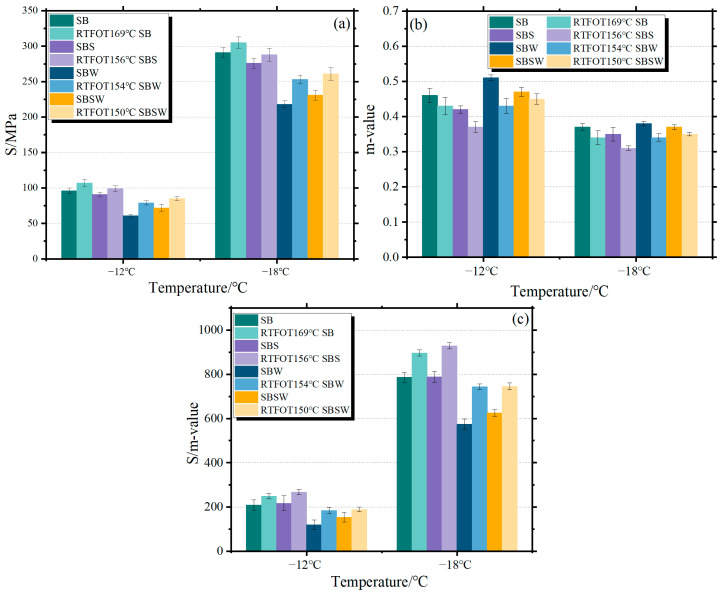
Rheological performance of modified BBs at low temperatures.

BBR test results at −12 °C and −18 °C for several types of bitumen are shown in Figure 4. The error bars represent the dispersion of the S/M and m-values for the same bitumen under multiple trials. In Figure 4a,c, it can be observed that regardless of the aging temperature, the performance trends of all specimens are consistent, with an increase in both S and S/m values, while the m value decreases. This suggests that while the stiffness and brittleness of the bitumen increase as a result of rapid aging, the low-temperature cracking resistance of SB and SBSW diminishes. Furthermore, both before and after RTFOT, the S of SB is larger than that of SBS, SBW, and SBSW; this shows that the modifiers increased the bitumen’s flexibility at low temperatures. Compared to the nonaged SBSW, the nonaged SBW exhibits a higher m value, as can be seen in Figure 4b. However, interestingly, after the specimens undergo RTFOT, the relationship between their m values becomes reversed. Due to the difficulty in evaluating the low-temperature performance of modified BB based on S or m values alone, the S/m ratio was used instead, which better reflects the deformation and stress relaxation capacity of the bitumen. It can be seen that the S/m values of SBSW are lower than those of SB and SBS at all test temperatures before and after aging. Under identical test temperatures, the incorporation of the Sasobit/WCO modifier leads to a substantial enhancement in the cracking resistance of SB, as indicated by the ranking of SBS > SB > SBSW > SBW in terms of S/m values.

The following factors may be responsible for the phenomena where Sasobit/WCO can marginally raise the m values of RTFOT-aged SB. Under the testing temperatures, Sasobit’s wax lattice architectures can significantly reduce the m-value while increasing creep stiffness. Meanwhile, WCO’s improved thermal-stress-relieving capabilities as a lightweight oil can productively offset the negative effects of Sasobit [45]. Under the combined action of Sasobit and WCO, SBSW has a certain creep stiffness and stress relaxation capacity. However, WCO experiences thermal breakdown at the 150 °C RTFOT test temperature, which reduces WCO’s moderating power. As a result, after RTFOT aging, the m-value of SBW with higher WCO concentration is marginally lower than that of SBSW.

### 3.4. Rheological Performance

#### 3.4.1. DSR Test Results

**Figure 5:**
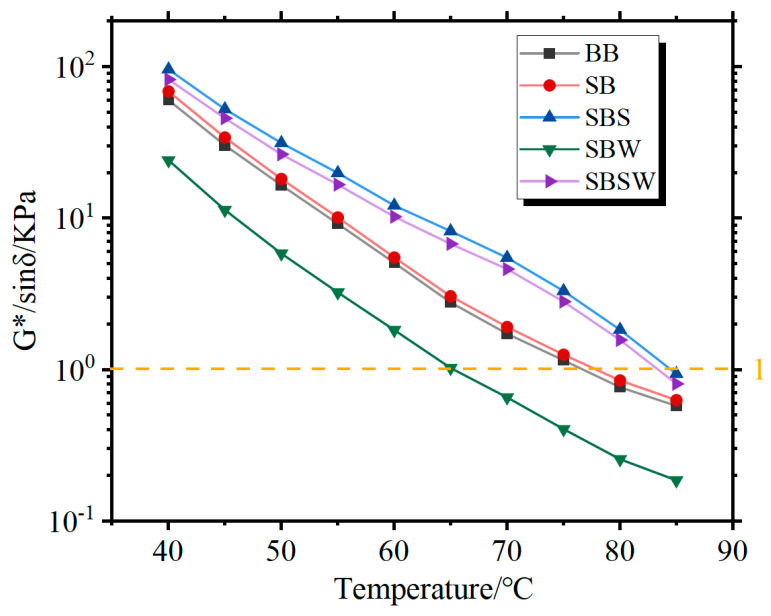
Rutting parameters of nonaged modified BBs.

The rutting factor (G*/sinδ) was used to evaluate the high-temperature stability of the modified BB [47]. The G*/sinδ values of unaged BBs are shown in Figure 5. For the same temperature, BBs with greater rutting factors have better rutting resistance. The temperature corresponding to a rutting factor of 1.0 kPa is often used to visualize the difference in bitumen performance. It can be seen that the high-temperature critical temperatures for unaged SBS, SBSW, SB, BB, and SBW are 84.8 °C, 83.4 °C, 76.5 °C, 76.2 °C, and 65.1 °C, respectively. This suggests that the Sasobit/WCO combination can significantly increase the SB’s high-temperature rutting resistance. Additionally, the G*/sinδ difference between unaged base BB and unaged SB is minimal at all sweep temperatures, which demonstrates that SBR has a negligible impact on base BB’s antirutting ability.

**Figure 6:**
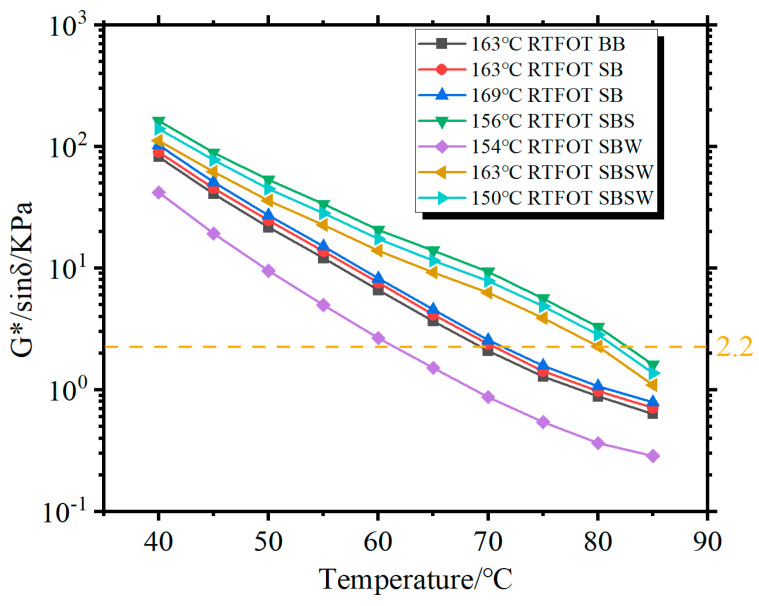
Rutting parameters of RTFOT-aged modified BBs.

The G*/sinδ values of all RTFOT-aged BBs are shown in Figure 6. It is evident that among all modified BBs, 156 °C RTFOT SBS has the greatest G*/sinδ in the entire temperature range, indicating that it has the best high-temperature rutting resistance capabilities. Bitumen aging becomes progressively more severe with increasing temperature, thus weakening its resistance to deformation at high temperatures, which can be shown by the fact that the 163 °C RTFOT-SBSW’s G*/sinδ is only second to 156 °C RTFOT-SBS and 150 °C RTFOT-SBSW. Additionally, the high-temperature critical temperatures for 150 °C RTFOT-SBSW, 163 °C RTFOT-SBSW, 169 °C RTFOT-SB, 163 °C RTFOT-SB, and 163 °C RTFOT-BB are 81.9 °C, 80.1 °C, 70.6 °C, 70.1 °C, and 69.7 °C. These can indicate that Sasobit/WCO addition and the lowering of the aging temperature both help RTFOT-SB’s resistance to rutting at high temperatures. It is important to note that the G*/sinδ discrepancies between the 163 °C RTFOT-BB, 157 °C RTFOT-SB, and 163 °C RTFOT-SB are small, which is consistent with the results of the unaged BB’s G*/sinδ.

#### 3.4.2. BBR Test Results

**Figure 7:**
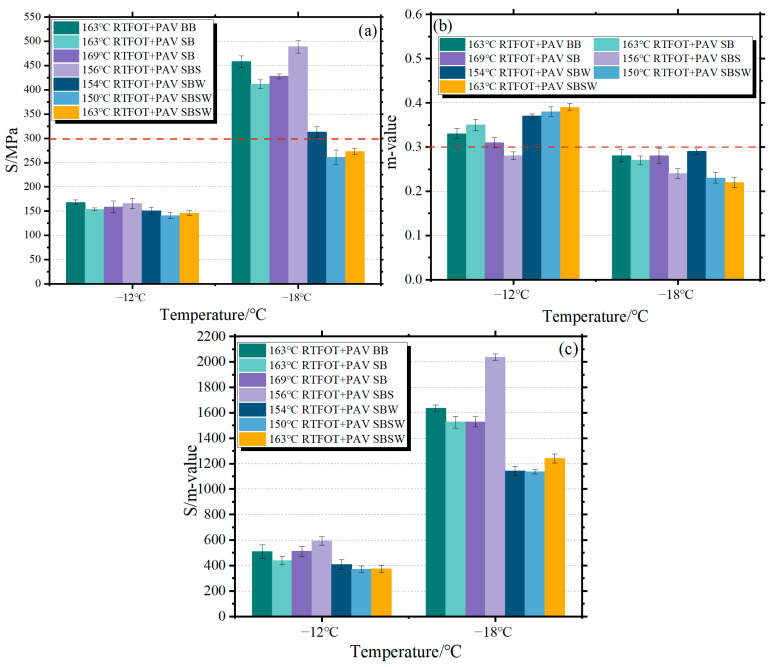
BBR results of PAV-aged BBs: (**a**) S, (**b**) m-value, (**c**) S/m-value.

The BBR test results of all PAV-aged modified BBs at −12 °C and −18 °C are depicted in Figure 7. It can be clearly seen that PAV-aged SBSW consistently has the lowest value of S among all modified BBs. This observation demonstrates that the utilization of Sasobit/WCO has the capacity to mitigate thermal stresses in SB, consequently reducing the susceptibility of SB pavement to thermal cracking. Furthermore, a decrease in the RTFOT aging temperature from 163 °C to 150 °C leads to a reduction in the S of PAV-aged SBSW. Nonetheless, concerning the m-value, except at −18 °C, the incorporation of Sasobit/WCO demonstrated an ability to increase the m-value of SB, suggesting a favorable influence of Sasobit/WCO on the stress relaxation characteristics of SB. Given the disparate effects of S and m-value on the modification of SB, the S/m value ratio is regarded as a pivotal evaluation parameter. At all testing temperatures, as seen in Figure 7c, the S/m-value of 150° CRT+PAVSBSW is lower than 169° CRT+PAVSB, indicating that SBSW has a greater low-temperature cracking resistance. According to the S/m-value results, Sasobit/WCO’s reduction in the short-term aging temperature and the synergistic impact of Sasobit/WCO have a favorable impact on the long-term thermal fracture resistance of SB.

### 3.5. Performance Grade (PG)

**Figure 8:**
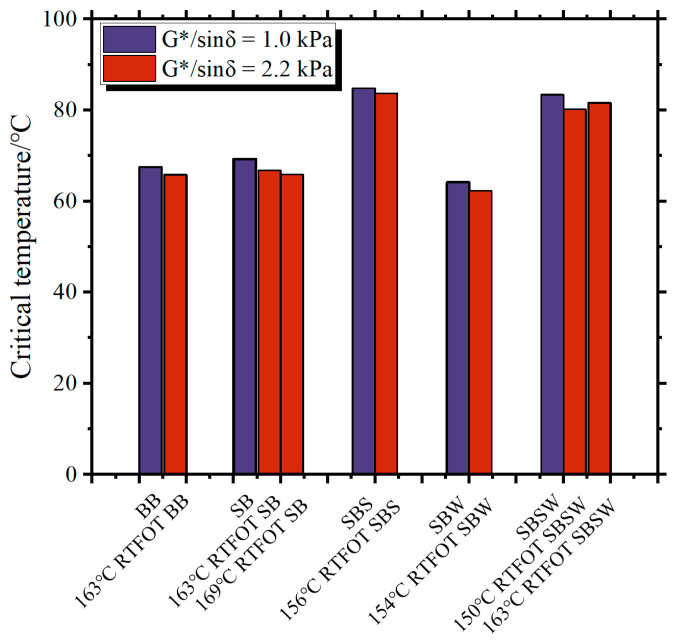
Critical temperatures of different BBs.

**Figure 9:**
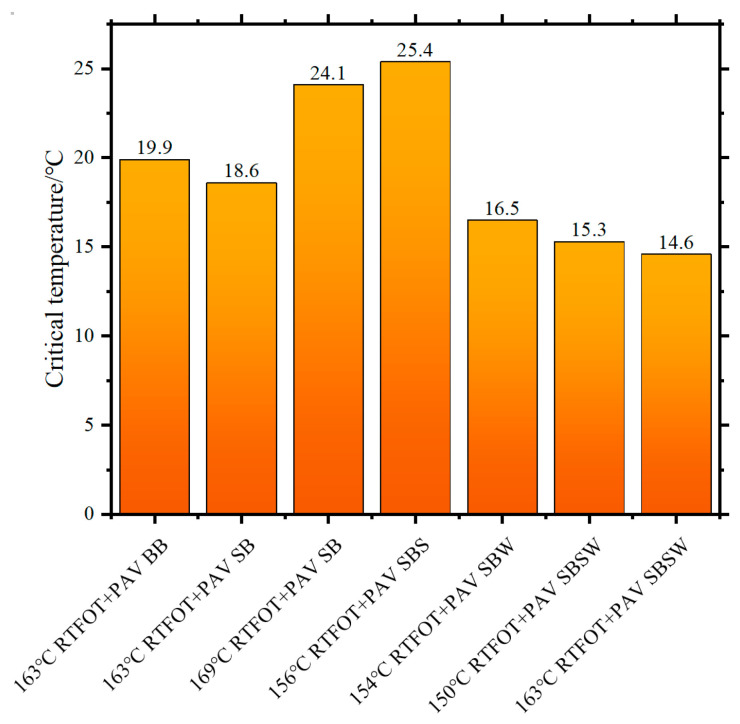
Critical temperatures of BBs for PAV-aged samples.

Figure 8 and Figure 9 present a comprehensive overview of the critical temperature outcomes associated with G*/sinδ values of 1.0 kPa and 2.2 kPa, as well as G*sinδ attaining 5000 kPa, respectively. These findings offer valuable benchmarks for the determination of the PG of bitumen binders within the context of scientific investigation [47]. The sequence of critical temperatures for G*/sinδ = 1.0 kPa in Figure 8, with SBS > SBSW > SB > BB > SBW, aligns closely with the softening point. Furthermore, the critical temperature differences between BB and SB are less than 1 °C both before and after RTFOT aging, demonstrating the limited impact of SBR on BB’s thermal stability.

By determining the critical temperature at which G*sinδ = 5000 kPa, it becomes possible to assess the fatigue crack resistance of modified BB after long-term aging. Greater fatigue crack resistance is achieved with lower critical temperatures. As seen in Figure 9, SBSW consistently exhibits the lowest critical temperature across all bitumen samples, regardless of whether the viscosity-reducing impact of Sasobit/WCO is taken into account. The result shows that SBSW has greater resistance to fatigue cracking than BB and SB. Additionally, the critical temperature ranking is BB > SB > SBSW at 163 °C This suggests that the inclusion of SBR polymer enhances the resistance of BB to fatigue cracking, and the supplementary incorporation of Sasobit/WCO amplifies this effect. It is important to note that the critical temperatures for 163 °C RTFOT+PAV SBSW and 150 °C RTFOT+PAV SBSW are quite close, indicating that the decrease in temperature has no impact on SBSW’s resistance to fatigue cracking.

According to the outcomes of the rheological property assessments, the PG for all bitumen specimens is detailed in Table 6. Notably, it is evident that under standard or equivalent short-term aging temperature conditions, the high-temperature PG of SBSW surpasses that of SB and BB by two grades. This finding indicates that the incorporation of Sasobit/WCO compounds can enhance the fatigue cracking and high-temperature rutting resistance of SB. In addition, SB and SBSW had the same grade for low-temperature PG, proving that Sasobit/WCO’s introduction did not weaken SB’s low-temperature PG.

## 4. Conclusions

In this study, the effects of Sasobit/WCO compounds on the physical, rheological, and aging properties of SBR bitumen were investigated using conventional performance tests, DSR, and BBR tests. The following are significant conclusions drawn from the test results:

(1) Compared to Sasobit and WCO, Sasobit/WCO can improve SB’s performance at high and low temperatures. The combination of Sasobit and WCO not only raises the softening point of SBRBBSB by 30.4 °C but also enhances its ductility at 5 °C, increasing it from 67.2 cm to over 150 cm.
(2) Before and after aging, the Sasobit/WCO compound can significantly improve the SB’s low-temperature anticracking performance. Adding Sasobit/WCO can considerably increase the SB’s short- and long-term aging resistances.
(3) The mixing temperature of the SB combination can be lowered by additives like Sasobit, WCO, and Sasobit/WCO. Sasobit/WCO in particular can significantly lower the preparation temperature by 19 °C.
(4) SBSW has better high-temperature PG than SB, regardless of the warm-mix effect produced by Sasobit/WCO, and Sasobit/WCO can boost SB’s resistance to high-temperature rutting and fatigue cracking. The high-temperature PG of SBSW surpasses that of SB and BB by two grades.

## 5. Recommendations for Future Work

Sasobit/WCO composite modified asphalt holds immense promise for the sustainable evolution of road construction. The primary emphasis of this study centered on assessing the impact of Sasobit/WCO on SBR-modified BB. Future investigations will encompass an expanded battery of performance tests on the bitumen mixture, facilitating a comprehensive evaluation of Sasobit/WCO’s influence on the SB mixture and a more robust demonstration of the BB test outcomes. In addition, future research efforts should concentrate on performance enhancement, environmental impact assessment, field applications, economic viability, and standardization.

## Figures and Tables

**Table 1:** Properties of base bitumen.

Parameter	Unit	BB	SB
Penetration (25 °C)	0.1 mm	71.9	73.1
Ductility (5 °C)	cm	5.6	67.2
Softening point	°C	47.3	49.1

**Table 2:** Basic indicators of SBR.

Properties	Unit	Results
Density (25 °C)	g/cm^3^	0.96
Styrene content	%	22.9
Organic acids	%	3.1
pH (25 °C)		5.8
Physical form (25 °C)		Milky fluid

**Table 3:** Physical indicators of Sasobit.

Properties	Unit	Results
Density (25 °C)	g/cm^3^	0.89
Viscosity (135 °C)	mPa. s	7.23
pH (25 °C)	-	5.8
Melting point	°C	103
Flash point	°C	287

**Table 4:** Physical properties of WCO.

Index	Unit	Test Results
Viscosity (25 °C)	mPa.s	127
Flash point	°C	296
Fire point	°C	323
Density	%	0.916

**Table 5:** The preparation temperatures of SB, SBS, SBW, and SBSW.

Bitumen	SB	SBS	SBW	SBSW
Mixing temperature (°C)	169	156	154	150
Compaction temperature (°C)	157	144	142	138

**Table 6:** PG of bitumen binders.

Bitumen Sample	PG
163 °C RTFOT+PAV BB	PG 64-22
163 °C RTFOT+PAVSB	PG 64-22
169 °C RTFOT+PAVSB	PG 64-22
156 °C RTFOT+PAVSBS	PG 76-22
154 °C RTFOT+PAVSBW	PG 64-22
150 °C RTFOT+PAVSBSW	PG 76-22
163 °C RTFOT+PAVSBSW	PG 76-22

## Data Availability

Data are contained within the article.
